# Words from spontaneous conversational speech can be recognized with human-like accuracy by an error-driven learning algorithm that discriminates between meanings straight from smart acoustic features, bypassing the phoneme as recognition unit

**DOI:** 10.1371/journal.pone.0174623

**Published:** 2017-04-10

**Authors:** Denis Arnold, Fabian Tomaschek, Konstantin Sering, Florence Lopez, R. Harald Baayen

**Affiliations:** 1 Quantitative Linguistics, Seminar für Sprachwissenschaft, Eberhard Karls Universität Tübingen, Tübingen, Germany; 2 Institut für Deutsche Sprache, Mannheim, Germany; Rijksuniversiteit Groningen, NETHERLANDS

## Abstract

Sound units play a pivotal role in cognitive models of auditory comprehension. The general consensus is that during perception listeners break down speech into auditory words and subsequently phones. Indeed, cognitive speech recognition is typically taken to be computationally intractable without phones. Here we present a computational model trained on 20 hours of conversational speech that recognizes word meanings within the range of human performance (model 25%, native speakers 20–44%), without making use of phone or word form representations. Our model also generates successfully predictions about the speed and accuracy of human auditory comprehension. At the heart of the model is a ‘wide’ yet sparse two-layer artificial neural network with some hundred thousand input units representing summaries of changes in acoustic frequency bands, and proxies for lexical meanings as output units. We believe that our model holds promise for resolving longstanding theoretical problems surrounding the notion of the phone in linguistic theory.

## Introduction

The invention of alphabetic writing systems has deeply influenced western reflection on language and language processing [1]. Just as letters make up written words, spoken words are assumed to consist of sequences of speech sounds (phones), the universal building blocks of language [2]. However, acoustic realizations of phones and words are known to be extremely variable within and across speakers. Nevertheless, it is generally accepted that the understanding of spoken words hinges on the identification of phones. In linguistics, psycholinguistics, and cognitive science, it is widely assumed that the only way in which the extreme variability in the speech signal can be dealt with is by funneling speech comprehension through abstract phone representations or feature bundles derived thereof [3, 4].

The validity of phones as central units of linguistic theory has not gone unchallenged [5]. It is well-known that phone recognition depends on surrounding phones. For instance, the difference between *p* and *k* is carried primarily by formant transitions in surrounding vowels [6] and the difference between postvocalic *p* and *b* is often indicated by the duration of the vowel [7]. Although recent years have seen the development of automatic speech recognition systems that eschew phones and phone inventories, such as the Deep Speech system [8], models of auditory comprehension in cognitive science and psycholinguistics build on phones as central theoretical units [9–12].

An unsolved problem for theories building on the phone as foundational unit for auditory comprehension is that in conversational speech, words tend to be uttered with substantially shortened forms. In English, *hilarious* can reduce to *hleres* [13], in Dutch, *natuurlijk* reduces to *tuuk* [14], and in German, *würden* is shortened to *wün* [15]. A survey of English spontaneous conversations [13] indicates that some 5% of words are spoken with one syllable missing, and that a little over 20% of words have at least one phone missing. It has been argued that reductions arise from stronger anticipatory coarticulation due to higher linguistic experience with these words [16, 17]. Importantly, when speakers do understand reduced forms such as *hleres*, the sound image that reaches awareness is not the reduced form, but the canonical citation form (*hilarious*) [18]. Thus, there is a large discrepancy between the signal that drives recognition and the word form available to conscious reflection [5]. Importantly, adding reduced forms to the lexicon tends not to lead to enhanced performance, as the number of improvements is typically offset by a similar number of deteriorations [13, 19].

Here, we present a novel cognitive model for the initial stage of auditory comprehension that does not build on the phone as theoretical unit, while addressing the comprehension of reduced forms in a principled way. Trained on a mere 20 hours of German spontaneous speech, this computational model correctly identifies 25% of the words presented in the experiment described below. Here, we also document that this accuracy is well within the range of human performance. Of theoretical importance is that the model does not make use of any representations for phones or phonic words. At the heart of the model is a ‘wide’ learning algorithm [20–22] that takes acoustic features as inputs and pointers to semantic vectors [23, 24] as outputs (henceforth lexomes).

## Materials and methods

### Materials

We used the GECO corpus v1.0 [25] for training our model as well as for sampling stimuli for our comprehension experiments. The corpus consists of spontaneous dialogues between 13 female speakers unknown to each other. In total, the corpus contains 20 hours of speech.

### Formalization of learning

Cognitive performance was modeled by means of supervised learning using the Rescorla-Wagner Eq (2) [26], a learning algorithm closely related to the perceptron [27] and adaptive learning in electrical engineering [28]. The Rescorla-Wagner learning rule predicts many aspects of both animal [29] and human learning [30, 31], and also predicts a wide array of findings in human lexical processing [32]. This learning rule fits well with the firing patterns observed for dopaminergic neurons [33] and may have evolutionary advantages in natural selection [34].

The Rescorla-Wagner equations estimate the association strengths (weights) on the connections between a set of input units C (with cardinality *k*), henceforth *cues*, and a set of output units O (with cardinality *n*), henceforth *outcomes*. After exposure to all training data, and having encountered all *k* cues and all *n* outcomes, the network is defined by a *k* × *n* matrix of connection weights. In the course of learning, the weight matrix will be smaller, as at a given point in time *t*, only a subset of cues and outcomes will have been encountered. For each learning event *L*_*t*_, *t* = 1, 2, …, *T*, weights are adjusted on the connections from the cues actually present in the input of that learning event, henceforth the *active* cues $C_{t}$ ($C_{t}\subseteq C$), to all the outcomes $O_{1,\ldots ,t}$ that have been encountered at least once during any of the learning events 1, 2, …, *t*, henceforth $O_{t}$ ($O_{t}\subseteq O$). The change effected in the weight from cue *c*_*i*_ to outcome *o*_*j*_ at learning event *t*, $\Delta w_{ij}^{t-1}$, which defines the weight at the end of learning event *L*_*t*_,

$$\begin{array}{c} w_{ij}^{\left(t\right)}=w_{ij}^{\left(t-1\right)}+\Delta w_{ij}^{\left(t-1\right)}, \end{array}$$ (1)

is given by the Rescorla-Wagner equations. Letting *I*_[*γ*]_ evaluate to 1 if condition *γ* is true, and to 0 otherwise, the change in weight is defined as follows:

$$\begin{array}{c} \Delta w_{ij}^{\left(t-1\right)}=\left\{\begin{array}{cc} 0 & \text{if}\;c_{i}∋C_{t},\\ \alpha_{i}\beta_{j}\;\left(\lambda -\sum_{m}\;{\text{I}}_{\left[c_{m}\in C_{t}\right]}w_{mj}^{\left(t-1\right)}\right) & \text{if}\;c_{i}\in C_{t}\wedge \;o_{j}\in O_{j},\\ \alpha_{i}\beta_{j}\;\left(0-\sum_{m}\;{\text{I}}_{\left[c_{m}\in C_{t}\right]}w_{mj}^{\left(t-1\right)}\right) & \text{if}\;c_{i}\in C_{t}\wedge \;o_{j}∋O_{j}\wedge o_{j}\in O_{1,\ldots ,t-1},\\ 0 & \text{otherwise}. \end{array}\right. \end{array}$$ (2)

In our calculations, *λ* is set to 1.0 and *α*_*i*_ *β*_*j*_ = 0.001 for all *i*, *j*. The first condition in Eq (2) concerns cues that are not in the input. Weights on efferent connections from such cues are left unchanged. The second condition applies to cues and outcomes that are both present in the learning event *t*. In this case, weights are strengthened. Furthermore, when many cues are present simultaneously in *t*, the magnitude of the increase tends to be reduced. The third condition handles the adjustments of the weights on connections from cues that are present in the input to outcomes that are not present, but that have been encountered previously during learning. Weights are now decreased, and the decrease will tend to be larger when more cues are present in the input. The fourth condition concerns associations between cues and outcomes that have not yet been encountered. Here, association weights remain unchanged.

### Acoustic cues

The Rescorla-Wagner learning rule requires discrete cues. Hence, an algorithm is required that derives a comparatively small number of discrete cues from the speech signal. The by-word Frequency Band Summaries (FBS) algorithm takes the speech signal of a word (as available in a speech corpus) as input, and derives discrete acoustic cues as follows.

First, the speech signal of a word is resampled to 16 kHz, using the function resamp() provided by the seewave (version 2.0.5) package for R [35, 36].

**Fig 1:**
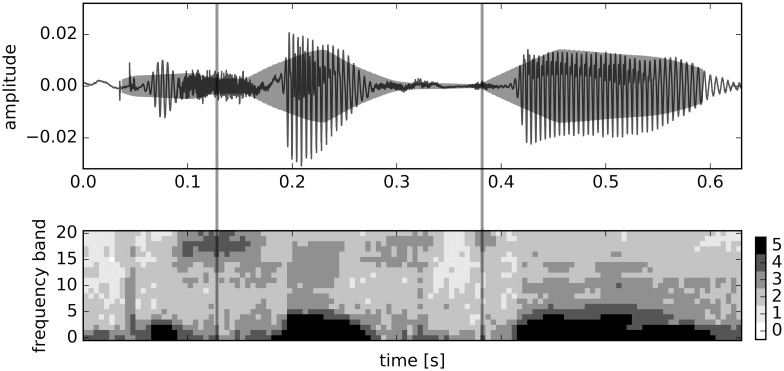
Oscillogram with Hilbert amplitude envelope for the German word *Geschichte* ‘history’ (top panel) and corresponding mel scaled spectogram (lower panel). Vertical lines represent the boundaries calculated from the minima in the Hilbert amplitude envelope. For this example, 21 FBS features are extracted for each of the three chunks of speech, resulting in a total of 63 FBS features for this word.

Second, the signal is partitioned into chunks using minima of the Hilbert amplitude envelope. The function env() provided by the seewave package [36] was used to compute the Hilbert amplitude envelope [37], with a Daniell kernel for the smooth with a kernel dimension of 800 [38]. Minima on the envelope were extracted with the function rollapply() provided by the zoo (version 1.7-13) package, evaluating whether the middle segment of a 1000 sample long window (62.5 ms) has the smallest value. In case this is true, a chunk boundary is positioned at this segment (see the top panel of Fig 1).

Third, for each chunk, we obtained FBS features as follows. We calculated the power spectrum using a window length of 5 ms without overlap, resulting in 64 frequency bands (0 to 8000 Hz). Subsequently, we transformed the power spectrum by means of a critical band analysis to 21 mel spectrum bands of equal width, resulting in a time by frequency matrix ***M***. A detailed description of the transformation can be found in [39] We used powspec() and audspec(), both provided by tuneR (version 1.3.1), to perform these steps. Intensities in these frequency bands were discretized by first taking the logarithm of ***M*** and applying the following equation (with *s* the number of discrete intensity values, 5 in the present study):

$$\begin{array}{c} M_{s}=\left\lceil \frac{s\{M-\min(M)\}}{\left|\min(M)-\max(M)\right|}\right\rceil \end{array}$$ (3)

The resulting discretized spectrum is exemplified in the lower panel of Fig 1.

Fourth, for each chunk, and for each of the 21 frequency bands in a given chunk, the information in this band is summarized. Thus, for a word with *N* chunks, there are *N* × 21 features. A feature summary consists of frequency band number, the first intensity value, the median of all values in the frequency band, the minimum and maximum intensity, the last intensity value, and chunk index. Thus, the FBS feature band1start1median2min1max4end2part1 specifies that in the lowest frequency band in the first chunk, the first intensity value is 1, the median in the whole frequency band is 2, the minimum is 1, the maximum is 4 and the final value is 2. Although transparent for the user, for the learning algorithm, an FBS feature is simply an identifier. FBS features are implemented in the AcousticNDLCoder [40] (version 1.0) package for R.

FBS features are inspired by the different receptive areas of the cochlea known to be responsive to variation in specific frequency ranges in acoustic signals [41]. Their cognitive conceptualization is that for a given frequency band, several neural cell assemblies respond to aspects of the temporal dynamics, such as maximum or minimum frequency reached. A specific FBS feature is therefore a proxy for the joint response of these lower-level cell assemblies.

**Fig 2:**
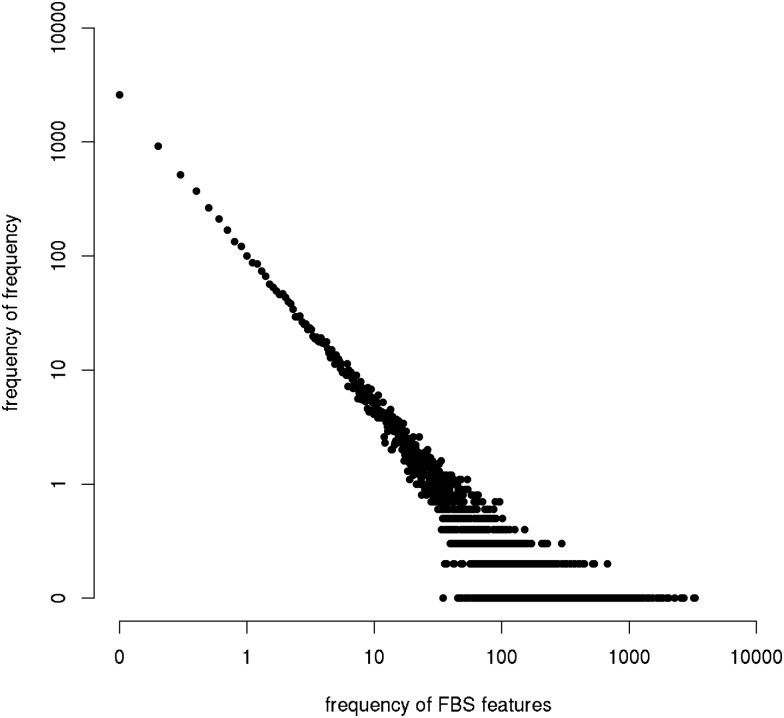
The frequency distribution of FBS features follows a power law with negative slope in the log-log plane.

For the speech in the GECO corpus, 78,814 different FBS features were extracted. The grouped frequency distribution of the FBS features follows a power law (Fig 2). An important property of FBS features is that they are robust to differences in speech rate, and that they do not require prior speaker normalization.

### Model-based predictors

A Rescorla-Wagner network with 78,814 FBS features as cues and 13,388 lexomes as outcomes was trained on the 246,625 word tokens in the order in which these appeared in the speech files of the GECO corpus. A word token in this case is every element in the transcriptions that is not enclosed by angled brackets “< >”. A lexome is a unique word token. Each word token contributed a learning event with FBS features extracted from the speech file using the word boundaries available in the corpus, and as outcome the word’s orthographic form, representing a pointer to that word’s location in semantic vector space. For each learning event, weights were updated according to the Rescorla-Wagner learning Eq (2), resulting in a weight matrix ***W*** characterizing the final state of the network.

Two predictors are derived from ***W***. First, the activation $a(O_{j})$ of outcome $O_{j}$ is defined as the sum of the afferent weights to $O_{j}$ originating from the active cues of word *ω*, $C_{\omega }$:

$$a\left(O_{j}\right)=\sum_{i}{\text{I}}_{\left[c_{i}\in C_{\omega }\right]}w_{ij}.$$

Presentation of the FBS features of a word stimulus $C_{\omega }$ to the network results in a distribution $A_{C_{\omega }}$ over the output layer of the network, with values that tend to fall within the interval [-1, 1]. The outcome (lexome) with the highest activation is selected as the model’s best candidate.

Second, the distribution of activations itself is also informative. In order to provide evidence for lexicality, the acoustic input should support at least some words, and thus there should be at least some values in the activation vector $A_{C_{\omega }}$ that are sufficiently different from zero. An activation vector with only values close to zero characterizes a situation where only unintelligible noise is perceived, and for which we expect the participants in our experiment to make a *no*-response. A *yes*-response can be made only when there is at least one strongly activated lexome. Due to the dense nature of lexical similarity space [42, 43], however, a given acoustic input will typically activate not just one lexome, but a range of lexomes. Thus, an activation vector for a clear signal will tend to have many non-zero values, providing support for lexicality and a *yes*-response. We use the L1-norm (absolute length) of the vector of lexome activations given active cues $C_{\omega }$,

$$\text{L1-norm}\left(C_{\omega }\right)=\sum_{j=1}^{n}\left|a\left(O_{j}\right)\right|,$$

to assess the amount of support for lexicality. An L1-norm close to zero is a strong indicator of lack of contact with the lexicon, and predicts fast rejections in identification tasks. Larger values of the L1-norm indicate good support for lexicality, and predict slow acceptance in identification. Acceptance is predicted to be slow because irrelevant but well-supported competitors slow identification decisions.

### Experiment

We performed an auditory comprehension experiment with a random sample of words from the GECO corpus as stimuli. Four groups of 10 native speakers of German (34 female, 6 male, mean age 23 years, sd 3.9, all right-handed, no report of speech or hearing disorder) volunteered to take part in the experiment. They were rewarded with 10 euro for their participation, or with course credit. None had any known auditory deficits. We applied the ethical standards of research with human participants at the faculty of arts of the University of Tübingen. The participants read and signed a standard informed consent form and were informed that they could stop participating in the experiment at any time and without any disadvantages for themselves. Responses were de-identified before the analysis. The researchers who analyzed the data were not involved in data collection. For statistical analysis, it is essential to retain (anonymized) identifiers for participants, so that participant can be entered as random-effect factor into a mixed effects regression analysis. Crucially, it is not possible from the data files that were analyzed (available for research purposes as part of the AcousticNDLCodeR package [40]) to reconstruct who the actual participants were.

**Fig 3:**
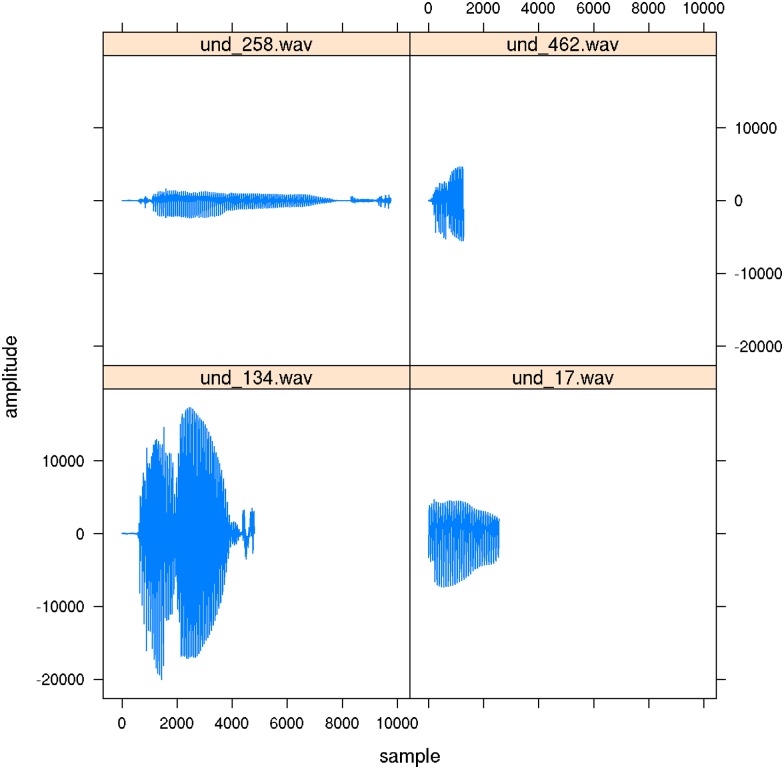
Examples of four realizations of German *und* (‘and’). Upper left: [ʊnt^h^], upper right: [ʊn], lowel left: [ʊnt^h^], lower right: [n].

Two sets of 500 stimuli were randomly sampled from the audio files in the GECO corpus v1.0, with as only restriction on the random sampling that the word type corresponding to the audio file occurs at least 11 times in the corpus. This is to ensure with a statistic probability that not all instances of a given word are in the test set and none are in the training set. The audio files sampled represent a total of 311 different word types, 40% of which are function words, with sample frequencies ranging from 1 (177 types, median frequency) to 52 (1 type). The five most frequent words in the stimulus set were *ja* (52), *ich* (48), *und* (38), *so* (34), and *das* (31). Fig 3 presents examples of the very different realizations of *und* ‘and’, with varying degrees of reduction. By sampling randomly, we ensured, first, that the experimental stimuli are representative of the tokens encountered in the corpus, and second, that the challenge of recognizing lower-frequency words is properly balanced by the challenge of recognizing highly reduced variants of high-frequency words.

In each experiment, 7 additional stimuli were used for a practice block. The audio files in the corpus were recorded at different volumes and presented to participants exactly as in the corpus, without any further normalization.

Two groups listened to the stimuli with Sennheiser headphones, and two groups listened to the stimuli with Creative loudspeakers placed at a distance of approximately 80 cm in a sound-attenuated booth.

Subjects were first asked to indicate, by pressing the *m* and *z* keys on a qwerty keyboard, whether they understood the word presented. We refer to this part of the task as the recognition task. Following their button press, subjects were requested to type in the word they had identified, or an ‘X’ if they had no idea of which word was presented. We refer to this part of the task as the dictation task. Button presses and time stamps for the button presses and onset of typing were collected with SR Research Experiment Builder version 1.10.1241 running on a Lanbox Lite computer running Windows 7 Professional. The experiment was self-paced. Short pauses were allowed after each block of 50 trials. The duration of the experiment varied between 40 and 60 minutes.

## Results and discussion

Recognition accuracy ranged from 40.6% to 98.8% (mean 72.6%), dictation accuracy ranged from 20.8% to 44.0% (mean 32.6%). Recognition accuracy and dictation accuracy were not correlated (*r* = 0.19, *t*_38_ = 1.17, *p* = 0.25). Dictation accuracy provides a more precise approximation of human identification performance than the self-reported recognition accuracy measure, which emerges as overly optimistic.

There was a significant difference in rating accuracy and reaction times for both tasks between listeners in the headphones condition and those in the loudspeaker condition. The listeners in the loudspeaker condition had a lower average dictation accuracy of 29.4% compared to 35.7% in the headphones condition. They also required significantly less time in both tasks, suggesting a speed-accuracy trade-off.

Identification accuracy, as gauged by dictation accuracy, was low compared to other studies. In [44], read speech and speech recorded from lectures elicited recognition rates ranging between 49.7% and 90% (depending on word duration). As lectures tend to require much more careful articulation than spontaneous face-to-face conversations, higher identification rates are to be expected. In [45], a range of speech registers was considered with as most informal register the Switchboard telephone conversations. For Switchboard, human identification accuracy for isolated word was at 88%. Given that speaking over the telephone to strangers also requires relatively careful pronunciation, the high accuracy is again unsurprising. Accuracy reported for highly spontaneous Dutch conversations is much lower, ranging from 88% for words with little reduction to 50% for highly reduced words [14]. As illustrated above in Fig 3, many of the words in our experiment are highly reduced.

The same audio files that were presented to participants were also presented to the wide learning network, resulting in, for each audio input, the lexome recognized (the lexome with the highest activation), the activation of the lexome recognized, and the L1-norm of the activation vector. For the two sets of 500 words presented to the two groups of participants in our experiment, average model accuracy was at 25.2%, with training on all but the sample of 1000 words used as stimuli in the experiments. The pairwise overlap in correctly identified words was similar and statistically indistinguishable for pairs of speakers and speaker-model pairings. In both the loudspeaker and headphones condition, subjects were present that performed with lower identification accuracy compared to our model (range loudspeaker condition 20.8–40.6%, range headphone condition 21.6–44.0%). The model still achieved an overall identification accuracy of 20% under the more stringent evaluation using leave-one-speaker-out cross-validation, evaluated on all in-vocabulary words.

**Fig 4:**
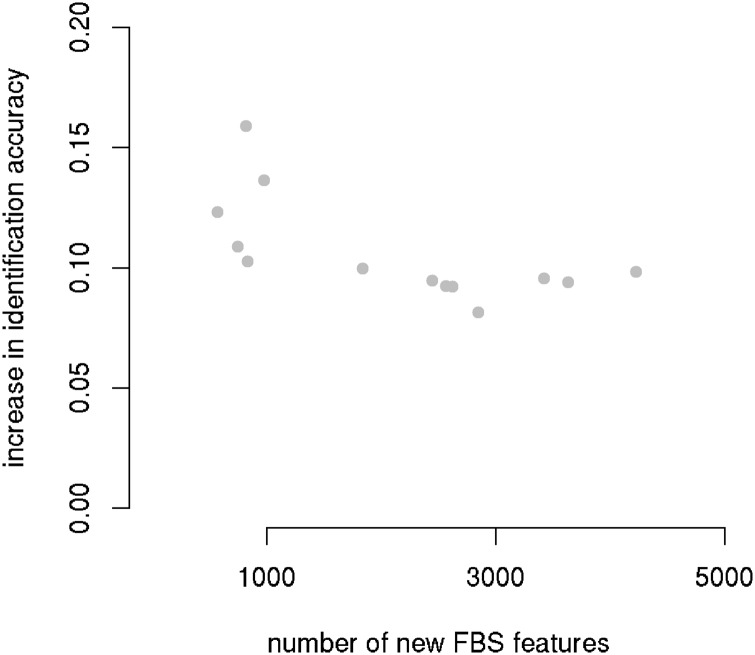
Speaker accommodation as a function of the number of novel FBS features in held-out speech. Each dot represents the increase in identification accuracy, comparing accuracy without and with training on the speech from the held-out speaker.

Just as listeners accommodate to their interlocutors, the model adapts to the speech of held-out speakers. This can be seen by comparing model performance on held-out speakers with and without training on these speakers’ audio. Performance with training on the speech of a given held-out speaker is superior to performance when the audio of that speaker is withheld from training, with an increase ranging from 8% to 17% (Fig 4). The greater the number of novel FBS features in the speech of the held-out speaker, the lower the improvement in accuracy is.

**Fig 5:**
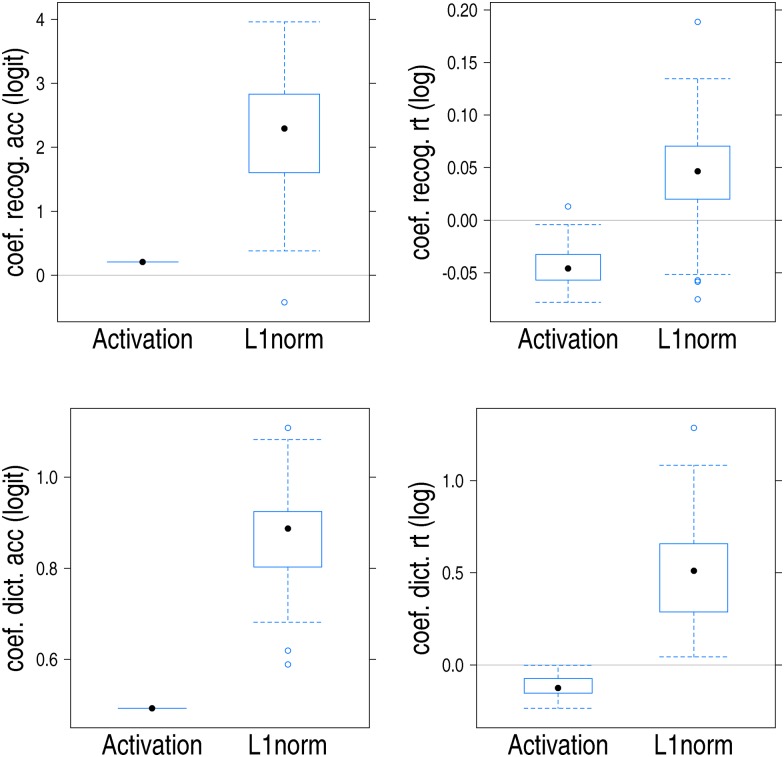
Boxplots for the estimated by-subject coefficients for LogL1norm and LogActivation in the recognition task (upper panels) and the dictation task (lower panels). Left: accuracy (on the logit scale); Right: latency (on the log scale). For recognition accuracy, the coefficients for LogActivation are those for the presentation over loudspeakers. For recognition latencies, the coefficients for both LogActivation and LogL1norm likewise pertain to presentation over loudspeakers.

Statistical evaluation of the recognition and dictation response variables was conducted with the help of the generalized additive mixed model (GAMM) [46, 47] with random intercepts for words and by-participant factor smooths for trial [48]. We used logistic GAMMs (and standard z-tests for evaluating coefficients) for modeling recognition scores and dictation accuracy, and Gaussian GAMMs (and standard t-tests) for modeling recognition and dictation latencies. Activations and L1-norms were log-transformed to reduce adverse effects of overly influential outliers, but as their distributions remained irregular, we restricted their effects to be linear. Recognition and dictation latencies were inverse transformed to make them amenable to Gaussian modeling. Table 1 documents the parametric coefficients of the models and associated statistics. Fig 5 visualizes subject variability around the estimates for the coefficients of LogActivation and LogL1norm. Complete documentation and data are available at the Open Science Framework at https://osf.io/cs9c2/.

**Table 1:** Coefficients, standard errors, test statistics, and p-values for the accuracy measures (upper part) and response latencies (lower table).

	Estimate	Std. Error	z value	Pr(>|z|)	Task
Intercept	-1.0601	0.5989	-1.7703	0.0767	recognition
LogL1norm	2.1008	0.2333	9.0048	<0.0001	recognition
PresentationMethodloudspeaker	-0.0773	0.7716	-0.1002	0.9202	recognition
LogActivation	-0.0885	0.1236	-0.7162	0.4739	recognition
PresentationMethodloudspeaker:LogActivation	0.2937	0.1159	2.5334	0.0113	recognition
Intercept	-1.4455	0.3164	-4.5684	<0.0001	dictation
PresentationMethodloudspeaker	-0.4944	0.1680	-2.9422	0.0033	dictation
LogL1norm	0.8698	0.1733	5.0184	<0.0001	dictation
LogActivation	0.4928	0.1166	4.2276	<0.0001	dictation
	Estimate	Std. Error	t value	Pr(>|t|)	Task
(Intercept)	7.3944	0.0774	95.5084	<0.0001	recognition
PresentationMethodloudspeaker	-0.1938	0.1055	-1.8378	0.0661	recognition
LogL1norm	-0.0246	0.0219	-1.1269	0.2598	recognition
LogActivation	-0.0108	0.0137	-0.7849	0.4325	recognition
PresentationMethodloudspeaker:LogL1norm	0.0667	0.0260	2.5640	0.0104	recognition
PresentationMethodloudspeaker:LogActivation	-0.0339	0.0152	-2.2306	0.0257	recognition
(Intercept)	6.1592	0.1384	44.4947	<0.0001	dictation
PresentationMethodloudspeaker	0.1772	0.1835	0.9655	0.3343	dictation
LogL1norm	0.5144	0.0561	9.1685	<0.0001	dictation
LogActivation	-0.1123	0.0249	-4.5132	<0.0001	dictation

A greater L1-norm (lexicality) predicted a greater probability of a *yes*-response in the recognition task across both presentation conditions, a longer recognition latency in the loudspeaker condition but not in the headphones condition, a greater dictation accuracy in both presentation conditions as well as a longer dictation latency in both conditions. When stimuli were presented over headphones, a greater L1-norm also predicted longer recognition latencies.

Activation was not predictive for recognition scores when stimuli were presented over headphones, but when loudspeakers were used for presentation, greater activation predicted higher recognition rates. A similar effect, but now robust across both presentation methods, was present for dictation accuracy. A greater activation also predicted shorter recognition latencies, but only when loudspeakers were used. A greater activation also predicted shorter dictation latencies, irrespective of presentation condition.

In summary, the wide learning model not only has an identification accuracy within the range of human performance, but several measures derived from the model’s weight matrix contribute to our understanding of the recognition and dictation response variables. Where significant, a greater activation affords greater accuracy and shorter response times, exactly as expected for a measure of bottom-up support. The L1-norm, as a general measure of evidence for lexicality, predicts both greater accuracy and longer response latencies: When the L1-norm is large, many potential lexical candidates are available. The resolution of this lexical conflict requires time, slowing yes responses but making them more accurate at the same time.

We note here that although identification accuracy of the model is low, at around 20–25%, the task of understanding isolated words taken from highly spontaneous speech is a hard one. This can be seen by inspecting performance of a standard industrial application, the Google Cloud Speech API, which correctly identifies 5.4% of the 1000 stimuli. The higher accuracy of wide learning is likely to be due to training on a much smaller corpus with far fewer speakers and a substantially more restricted vocabulary. At the same time, the GECO corpus likely offers many more instances of highly reduced words than the materials on which the Google Cloud Speech API is trained.

### Discussion

These results have far-reaching implications. Under optimal learning conditions, the initial stages of human processing of speech input may be far less complex than assumed by current state-of-the-art computational models in cognitive psychology [9, 10]. Importantly, the congruency of model predictions and native speaker performance strongly suggests that the speech signal of isolated words extracted from free spontaneous speech is rarely sufficient for complete identification, not for humans and not for computational models of human language processing. This in turn highlights the importance of (e. g., word *n*-gram) language models [49, 50] and clarifies why listeners are so exquisitely sensitive to non-linguistic cues for auditory comprehension (see also [51]), ranging from puffs of air [52] to symbols of national identity [53].

An important advantage of the wide learning approach adopted here is that it is not necessary to maintain a list of the many pronunciation variants (as in [11, 12]) that are part of the recognition problem. For example, the English word *until* appears with 10 different transcriptions in the Buckeye corpus [13], and such examples can easily be multiplied for other languages [54] (see Fig 3 for German). Models in which recognition takes place through matching with word forms either have to add countless reduced forms to their lexicon of canonical forms, or they have to devise ways in which mismatches such as between *tě* and its canonical form *until* are discounted. In current cognitive models of auditory comprehension, reduced forms are either ignored during model building and model evaluation [3, 9, 10] or failure of recognition of reduced forms is tolerated as random performance error [11]. These issues simply do not arise in our model.

**Fig 6:**
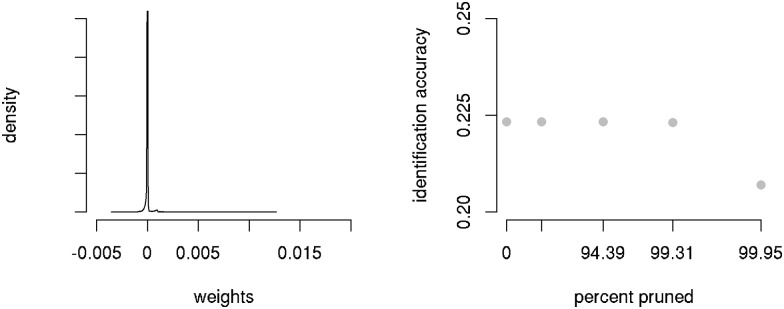
Left: Distribution of weights of afferent connections of *Geschichte*. Right: Identification accuracy calculated across the full data set for varying degrees of pruning.

Interestingly, the number of connection weights actually required for wide learning is only a fraction of the maximal network size. Since the distribution of connection weights is characterized by a majority of weights with values very close to zero (Fig 6), a dense matrix with 78,814 × 13,388 connection weights can be pruned down to a sparse matrix with 99.31% of the original connections removed. This mirrors connection pruning in human cortical development after the age of 14 [55, 56]. Importantly, pruning leaves accuracy unaffected. With only an average of 38 afferent connections to an output unit, the model respects estimates from neurobiology for single-cell receptivity [57, 58]. In the wide learning approach pursued here, the importance of non-acoustic cues for auditory comprehension can be accounted for under the simple assumption that visual and tactile input units are connected to lexomes in similar ways as FBS features. As for FBS to lexome connections, most weights on the connections from these visual and tactile input units to the lexomes will either be zero or driven close to zero by error, and hence will be pruned. But those non-auditory input units that are actually discriminative for auditory comprehension will remain present and will help boost comprehension of the acoustic signal.

Whether cognitive models of auditory comprehension can completely dispense with the phone as representational unit remains an open question. The phenomenon of categorical perception [59] and infants’ sensitivity to transitional probabilities [60] are pieces of evidence that are generally taken to support the cognitive validity of phones and auditory word representations. However, these phenomena have been shown [20] to follow straightforwardly from the present wide learning approach. Evaluation of the substantial literature on the cognitive reality of the phone is complicated by the acquired skill of sounding out letters that comes with literacy in alphabetic scripts.

We have demonstrated that it is possible to approximate auditory word recognition without phone representations, thus providing a radically new cognitive approach to the initial stage of speech recognition. Whether this approach can be extended to the recognition of continuous speech and compete with current standards in automatic speech recognition systems [61] is an open question that we are currently investigating. A related issue is whether phone-like units are essential for cognitive models of speech production. Given recent advances in speech technology using artificial deep neural networks [62], more may be possible without phones than current cognitive models would leave one to believe.
